# Intragroup Stigma Among Men Who Have Sex with Men: Data Extraction from Craigslist Ads in 11 Cities in the United States

**DOI:** 10.2196/publichealth.4742

**Published:** 2016-02-05

**Authors:** Tamar Goldenberg, Dhrutika Vansia, Rob Stephenson

**Affiliations:** ^1^ School of Nursing Department of Health Behavior and Biological Sciences and the Center for Sexuality and Health Disparities University of Michigan Ann Arbor, MI United States; ^2^ Rollins School of Public Health Hubert Department of Global Health Emory University Atlanta, GA United States

**Keywords:** stigma, online, MSM, Craigslist, sex ads

## Abstract

**Background:**

Gay, bisexual, and other men who have sex with men (MSM) regularly experience homophobic discrimination and stigma. While previous research has examined homophobic and HIV-related intergroup stigma originating from non-MSM directed at MSM, less is known about intragroup stigma originating from within MSM communities. While some research has examined intragroup stigma, this research has focused mostly on HIV-related stigma. Intragroup stigma may have a unique influence on sexual risk-taking behaviors as it occurs between sexual partners. Online sexual networking venues provide a unique opportunity to examine this type of stigma.

**Objective:**

The purpose of this study is to examine the presence and patterns of various types of intragroup stigma represented in Men Seeking Men Craigslist sex ads.

**Methods:**

Data were collected from ads on Craigslist sites from 11 of the 12 US metropolitan statistical areas with the highest HIV/AIDS prevalence. Two categories of data were collected: self-reported characteristics of the authors and reported biases in the ads. Chi-square tests were used to examine patterns of biases across cities and author characteristics.

**Results:**

Biases were rarely reported in the ads. The most commonly reported biases were against men who were not “disease and drug free (DDF),” representing stigma against men living with HIV or a sexually transmitted infection. Patterns in bias reporting occurred across cities and author characteristics. There were no variations based on race, but ageism (mostly against older men) varied based on the ad author’s age and self-reported DDF status; bias against feminine gender expression varied based on self-reported sexual orientation; bias against “fat” men varied by self-reported DDF status; bias against “ugly” men varied by a self-report of being good-looking; and bias against people who do not have a DDF status varied based on self-reported HIV status and self-reported DDF status.

**Conclusions:**

Despite an overall low reporting of biases in ads, these findings suggest that there is a need to address intragroup stigma within MSM communities. The representation of biases and intragroup stigma on Craigslist may result from internalized stigma among MSM while also perpetuating further internalization of stigma for men who read the sex ads. Understanding patterns in the perpetuation of intragroup stigma can help to better target messages aimed at making cultural and behavioral shifts in the perpetration of intragroup stigma within MSM communities.

## Introduction

Experiencing discrimination and stigma may have negative physical and mental health consequences for gay, bisexual, and other men who have sex with men (MSM). Research examining how discrimination among MSM is experienced has addressed two pervasive and often interacting forms of stigma: discrimination based on actual or perceived sexual orientation [1,2] and discrimination based on actual or perceived HIV status [3-5]. Experiences of homophobic or HIV-related stigma have been linked with increased suicide ideation [6-8], depression [6-9], substance use [10-13], and HIV risk [10,12-15] among MSM.

Another form of stigma that MSM may experience is intragroup stigma, defined as stigma within communities of MSM. Intragroup stigma may result from either the internalization of homophobic stigma among MSM or the heterogeneity of the MSM community. Communities of MSM contain a range of individual characteristics, such as race, age, HIV serostatus, etc, which could act as the basis for the generation of stigma within the MSM community. Research has examined how HIV stigma has been perpetuated among MSM; this type of intragroup stigma can lead to a rift or fracture within MSM communities with divisions based on HIV serostatus [16-18]. Intragroup stigma among MSM may also exist based on other characteristics, such as sexual orientation, race, class, gender identity, and body size; however, these other forms of possible stigma have received less research attention. Intragroup stigma among MSM is important to examine because it may have a different influence on health than intergroup stigma. Unlike intergroup stigma, intragroup stigma can be perpetuated by romantic and/or sexual partners, which may have implications for sexual risk and the negotiation of sexual encounters.

A useful source for examining intragroup stigma among MSM and between sexual partners is through Internet-based sex-seeking websites and apps. Online sex-seeking has become increasingly popular [19,20]; an estimated 40% of MSM in the United States have used the Internet to look for a sex partner [21-25]. Research suggests that MSM who have met their partners online report more sex partners [23,24,26,27], a higher prevalence of condomless anal intercourse [24,27,28], and a higher prevalence of sexually transmitted infections (STIs) [23,24,29-31]. However, research examining men seeking sex on Craigslist, a classified advertisements website, also states that the impact of online sex-seeking on MSM’s sexual risk-taking behaviors depends on the number of ads posted and the success of those ads [32]. The Internet may also have some protective factors for sexual risk-taking, such as increased negotiation around sex [33-37]. The online environment allows men seeking sex to negotiate location and type of sex and enables disclosure of information, including serostatus, prior to meeting. In a study by Grov et al, men who met their most recent sex partner online were more likely to disclose their HIV status compared with men who had met their most recent partner at other public places [25]. One reason for this increased negotiation may be the anonymity of meeting online partners. However, the anonymity, invisibility, and lack of eye contact inherent in online interactions may also result in online disinhibition, allowing those seeking sex online to say things that they would not say face-to-face [38-40], including discriminatory or stigmatizing statements. In this study, we explore whether the authors of sex ads report biases in their ads as a measure of the presence of stigma internal to the MSM community. Understanding the presence and forms of internal stigma in sex ads has the potential to inform messages aimed at risk prevention and stigma reduction among those seeking sex in online forums.

## Methods

Data were collected from 11 of the 12 metropolitan statistical areas (MSAs) with the highest HIV/AIDS prevalence in the United States, ranked by the Enhanced Comprehensive HIV Prevention Planning (ECHPP) project [41]. We chose MSAs with a high prevalence of HIV/AIDS to understand the presence and possible implications of intragroup stigma. The 12 MSAs include the cities of New York (New York), Los Angeles (California), Washington (District of Columbia), Chicago (Illinois), Atlanta (Georgia), Miami (Florida), Philadelphia (Pennsylvania), Houston (Texas), San Francisco (California), Baltimore (Maryland), Dallas (Texas), and San Juan (Puerto Rico). No Craigslist site exists for San Juan, so data were not collected. Data were extracted from ads on the Men Seeking Men section of the Craigslist sites from each of the cities.

Data collection was performed consecutively over 11 days (October 8, 2013 through October 18, 2013) with data collected from 1 city per day. After 2 data analysts developed a codebook with a list of variables for data extraction, they coded the first 50 ads for testing. Once the codebook was tested and finalized, a data analyst used the codebook to extract the data from the remaining ads. To minimize bias, data were collected from the first 200 ads listed before 2:30 PM (a randomly selected time) in each city’s time zone, standardizing the time of day for which data were collected across cities. Ads that were not looking for sex (eg, ads selling sex toys) or where couples created an ad together were excluded. This allowed for the correct identification of author characteristics. The total sample size included 2200 sex ads (200 per city). No identifying information was collected, and there was no interaction between the data collector and the subjects. Data were extracted from ads, entered into an Excel (Microsoft Corp) spreadsheet, and imported into STATA version 13.1 (StataCorp LP) for analysis.

We collected two types of variables: self-reported characteristics of the ad authors and reported biases in the ads. Domains not mentioned were coded as such in the data set. Self-reported characteristics included race/ethnicity (white, black, Latino, Asian, other); age (entered as a continuous variable and later categorized into age groups 18-25, 26-35, 36-45, 46 and above); sexual orientation (homosexual, heterosexual, bisexual); HIV status (negative, positive); self-reported “disease and drug free” status (“DDF,” “clean,” “healthy”); and self-reported physical appearance (“good looking,” “not good looking”). We use the terms “DDF,” “clean,” and “healthy” throughout this paper because those are the terms used by the sex ad authors. Since some characteristics were present in very few ads, categories were combined when analyzing the data. For characteristics, the race categories were combined to include only white, black, and other, and “DDF,” “clean,” and “healthy” were combined in one DDF status category.

Biases were defined as an ad in which the author specifically reported not wanting a characteristic in a sex partner or an ad that used stigmatizing language. The ad had to contain language stating “no X” or “X only,” with X representing a specific characteristic. For example, an ad was coded as including a bias if it included language such as “no HIV positive guys” or “white men only.” The following biases were collected: racism (saying no to black/Latino/Asian/other partners), ageism (saying no to a particular age group/range), weightism (saying no to “fat” or “underweight” men), heightism (saying no to tall or short men), transphobia (saying no to transgender people), physical appearance (saying no “ugly” men), gender expression (saying no “feminine” men or “no femmes”), HIV stigma (saying no “positive” men), and DDF status (saying must be “DDF,” “clean,” or “healthy”). DDF status was included as a bias because the terms “DDF,” “clean,” and “healthy” were considered stigmatizing language [34,42]. When a bias was present, it was entered into the codebook as a 1, and when it was not present it was entered as a 0. Data were analyzed using chi-square tests to determine variation in the demographic characteristics and biases across the 11 cities and across the demographic characteristics. Fisher exact tests were used when a demographic characteristic or bias was present fewer than 5 times. The alpha denoting significance was .05.

##  Results

### Sample Characteristics

The self-reported characteristics of the ad authors are described in Multimedia Appendix 1. The majority of the ads had minimal information about author characteristics. Among ads that contained race or ethnicity (853/2200, 38.77%), 63.4% (541/853) of the authors were white, 17.1% (146/853) were black, and 19.5% (166/853) were of another race (including Latino, Asian, and those who reported as other). Reporting of race was significantly different across the 11 cities (*P*<.001), with Baltimore representing the highest percentage of authors self-reporting race (104/200, 52.0%). Among those who self-reported race, Philadelphia represented the highest percentage of authors identifying as white (46/57, 80.7%). Miami and Los Angeles had a smaller percentage of ads with authors self-reporting as white (Miami, 23/48, 47.9%; Los Angeles, 49/98, 50.0%), with more of the authors in these cities identifying as Latino (Miami, 16/48, 33.3%; Los Angeles, 35/98, 35.7%). Most ads reported age (1991/2200, 90.50%), and age reporting in ads varied significantly across the cities (*P*=.003). Among ads reporting age, the modal age group of the authors was 26 to 35 years (689/1991, 34.61%), with Chicago representing the highest percentage of ads in this age group (80/187, 42.8%). Sexual orientation was not reported in the majority of ads; of those that did report sexual orientation (174/2200, 7.91%), 77.6% (135/174) of the authors reported being bisexual, 18.4% (32/174) heterosexual, and 4.0% (7/174) homosexual. Reporting of sexual orientation varied significantly across the cities (*P*<.001), with 16.0% (23/200) mentioning sexual orientation in New York and only 2.5% (5/200) mentioning it in Houston. Self-reports of HIV status were also low, with 86.50% (1903/2200) not mentioning their status. Among the ads that did mention self-reported HIV status (297/2200, 13.50%), 97.0% reported a negative serostatus. These reports of HIV status varied significantly across the 11 cities (*P*<.001); only 9.0% (18/200) of ads in New York self-reported HIV status compared to 26.0% (52/200) in Los Angeles. In addition, among men who did report HIV status, ads in Baltimore (n=20) were more likely to report a positive HIV status (3/20, 1.5%) compared with other cities. Among ads that contained self-reported DDF status (698/2200, 31.73%), 85.8% of authors reported being “DDF,” 13.9% reported being “clean,” and 0.3% reported being “healthy.” There were significant variations in DDF status across the cities (*P*<.001) with Houston representing the highest percentage of ads with authors describing themselves as “DDF,” “clean,” or “healthy” (73/200, 36.5%). The percentage of ads that contained reports of physical appearance (368/2200, 16.73%) varied significantly across the cities (*P*<.001) with Los Angeles and San Francisco representing the highest percentages of authors who reported being “good looking” (23.5% in both cities).

### Reported Biases

#### Overview of Biases

Overall, there were very few explicit reports of biases. Bias against men who are not “DDF” was the mostly commonly reported, with 24.55% (540/2200) of ads mentioning the need for a “DDF,” “clean,” or “healthy” partner. There were also more biases against physical appearance than most other biases with 4.36% (96/2200) of ads containing bias against “ugly” men. Weightism, which almost exclusively comprised bias against “fat” men, was reported in 2.32% (51/2200) of ads. Bias against gender expression, comprising bias against “feminine” men, was reported in 1.9% of the ads. Among ads with ageist biases (34/2200, 1.55%), most reports were against older men (32/34, 94.1%). There was very little racial bias reported (7/2200, 0.32%); these biases were reported against white men (n=4) and black men (n=3). Homophobia was the lowest reported bias with only 1 ad (1/2200) expressing bias against homosexual men (0.05%). No ads contained reports of bias against height, transgender people, or HIV status.

#### Variations in Biases by City

Variations in reported biases by city are presented in Tables 1-3 and reported by region (Northeast, South, and Midwest/West). Variations in self-reported biases by city were only significant for 3 variables: bias against physical appearance (*P*<.001), ageism (*P*=.03), and bias against men who are not “DDF” (*P*<.001). Bias against physical appearance was highest in ads from Los Angeles (24/200, 12.0%) and lowest in ads from Baltimore (4/200, 2.0%). Out of 11 cities, 9 contained ads with ageism; no ageist ads were present in New York and Washington. Among ads containing ageism, ageist bias was most present in Los Angeles with 5.0% (10/200) of ads reporting ageism overall and 4.5% (9/200) of ads reporting ageism directed at older men. Bias against men who were not “DDF,” “clean,” or “healthy” was highest in Dallas (61/200, 30.5%) and lowest in Philadelphia (33/200, 16.5%).

**Table 1:** Biases stratified by Northeastern cities (N=800).^a^

Biases (*P* value)^b^	MSAs with the highest HIV prevalence in the United States—Northeast				
	Baltimore n (%)	New York n (%)	Philadelphia n (%)	Washington n (%)	Total n (%)
Racism (*P*=.43)	2 (1.0)	1 (0.5)	0 (0.0)	0 (0.0)	3 (0.4)
Ageism (*P*=.03)	3 (1.5)	0 (0.0)	3 (1.5)	0 (0.0)	6 (0.8)
Weightism (*P*=.10)	7 (3.5)	2 (1.0)	2 (1.0)	2 (4.0)	13 (1.6)
Physical appearance (*P*<.001)	4 (2.0)	10 (5.0)	9 (4.5)	6 (3.0)	29 (3.6)
Gender expression (*P*=.83)	5 (2.5)	3 (1.5)	2 (4.0)	3 (1.5)	13 (1.6)
Homophobia (*P*=.44)	0 (0.0)	0 (0.0)	1 (0.5)	0 (0.0)	1 (0.1)
DDF status (*P*=.001)	55 (27.5)	48 (24.0)	33 (16.5)	55 (27.5)	191 (23.9)

^a^n=200 in each city.

^b^ *P* value is based on comparisons among all 11 cities.

**Table 2:** Biases stratified by Southern cities (N=800).^a^

Biases (*P* value)^b^	MSAs with the highest HIV prevalence in the United States—South				
	Atlanta n (%)	Dallas n (%)	Houston n (%)	Miami n (%)	Total n (%)
Racism (*P*=.43)	1 (0.5)	1 (0.5)	1 (0.5)	1 (0.5)	4 (0.5)
Ageism (*P*=.03)	1 (0.5)	1 (0.5)	3 (1.5)	4 (2.0)	9 (1.1)
Weightism (*P*=.10)	0 (0.0)	5 (2.5)	6 (3.0)	7 (3.5)	18 (2.3)
Physical appearance (*P*<.001)	6 (3.0)	11 (5.5)	7 (3.5)	5 (2.5)	29 (3.6)
Gender expression (*P*=.83)	2 (1.0)	5 (2.5)	3 (1.5)	4 (2.0)	14 (1.8)
Homophobia (*P*=.44)	0 (0.0)	0 (0.0)	0 (0.0)	0 (0.0)	0 (0.0)
DDF status (*P*=.001)	40 (20.0)	61 (30.5)	52 (26.0)	46 (23.0)	199 (24.9)

^a^n=200 in each city.

^b^ *P* value is based on comparisons among all 11 cities.

**Table 3:** Biases stratified by Midwestern and Western cities (N=600).^a^

Biases (*P* value)^b^	MSAs with the highest HIV prevalence in the United States—Midwest and West			
	Chicago n (%)	Los Angeles n (%)	San Francisco n (%)	Total n (%)
Racism (*P*=.43)	0 (0.0)	0 (0.0)	0 (0.0)	0 (0.0)
Ageism (*P*=.03)	4 (2.0)	10 (5.0)	5 (2.5)	19 (3.2)
Weightism (*P*=.10)	9 (4.5)	3 (1.5)	6 (3.0)	18 (3.0)
Physical appearance (*P*<.001)	8 (4.0)	24 (12.0)	6 (3.0)	38 (6.3)
Gender expression (*P*=.83)	2 (1.0)	4 (2.0)	7 (3.5)	13 (2.2)
Homophobia (*P*=.44)	0 (0.0)	0 (0.0)	0 (0.0)	0 (0.0)
DDF status (*P*=.001)	45 (22.5)	49 (24.5)	56 (28.0)	150 (25.0)

^a^n=200 in each city.

^b^ *P* value is based on comparisons among all 11 cities.

####  Variations in Biases by Sample Characteristics

Variations in biases by author characteristics are presented in Table 4. We found no statistically significant variation in biases based on the author’s race; however, there was variation on at least one reported type of bias for all other characteristics. Although more than 98.08% (2166/2200) of all ads across age groups contained no reports of ageism, reported ageism varied significantly by the age of the ad author (*P*=.006); 2.9% (11/381) of ads authored by those aged 18 to 25 years contained ageism compared to those aged 36 to 45 years (1/537, 0.2%) and aged 46 years and older (5/384, 1.3%). Only men in the 26 to 35 years age group reported ageism directed at younger men (n=2). Ageism also varied based on DDF status. Ads authored by men who self-report as “DDF” were more likely to contain ageism (13/698, 1.9%) compared to those authored by men who did not mention their DDF status (*P*=.04).

Bias against feminine men varied significantly by the sexual orientation of the ad author (*P*<.001). Although 98.09% (2158/2200) of the ads across authors of all sexual orientations contained no bias against feminine men, 6.7% (9/135) of ads authored by bisexual men and 6.3% (2/32) of ads authored by straight men contained bias against feminine men. Men who identified as homosexual reported no bias against feminine men.

Bias against men who are not “DDF” varied by HIV status (*P*<.001) and self-reported DDF status (*P*<.001). Men who reported a negative HIV serostatus were more likely to report bias against men who are not “DDF,” “clean,” or “healthy” (96/288, 33.3%) when compared with men who report a positive HIV serostatus (2/9, 22.2%) or men who did not mention serostatus (532/1903, 27.96%). Among men who reported a DDF status, 39.4% (275/698) reported bias to be with men who report a DDF status. Among men who do not report a DDF status, only 17.64% (265/1502) report a bias for a partner who is “DDF,” “clean,” or “healthy.”

Weightism varied significantly by the DDF status of the author (*P*=.04); 3.2% (22/698) of ads authored by men who identify as “DDF” contained weightism compared to 1.9% (29/1502) of ads authored by men who did not report their DDF status. Bias based on physical appearance varied significantly by the author’s self-report of being “good looking” (*P*<.001). Men who report being “good looking” are more likely to report bias against “ugly” men (44/368, 12.0%) compared to men who do not mention physical appearance (52/1832, 2.84%). The difference in reports of homophobic bias by age group was statistically significant (*P*=.05), but this was not a substantial finding because only 1 ad reported homophobic bias and this author did not mention his age.

**Table 4:** Variations in biases by author characteristics for all cities (N=2200).

Characteristics of ad authors		Biases							
		Racism	Ageism	Weightism	Physical appearance	Gender expression	Homophobia	DDF	Total n
**Race, ** ***P*** **value**		.11	.87	.06	.11	.09	.99	.79	
	White, n (%)	2 (0.4)	4 (0.7)	10 (1.9)	23 (4.3)	7 (1.3)	0 (0.0)	125 (23.0)	541
	Black, n (%)	0 (0.0)	4 (2.7)	6 (4.1)	1 (0.7)	7 (4.8)	0 (0.0)	35 (24.0)	146
	Other, n (%)	0 (0.0)	3 (1.8)	5 (0.9)	11 (2.0)	5 (0.9)	0 (0.0)	39 (7.2)	166
	Not mentioned, n (%)	2 (0.3)	23 (1.8)	30 (2.2)	61 (4.5)	23 (1.7)	1 (0.1)	275 (25.1)	1347
**Age,** ***P*** **value**		.35	.006	.85	.09	.10	.049	0.88	
	18-25, n (%)	0 (0.0)	11 (2.9)	10 (2.6)	20 (5.3)	12 (3.2)	0 (0.0)	96 (25.2)	381
	26-35, n (%)	0 (0.0)	17 (2.5)	17 (2.5)	39 (5.7)	17 (2.5)	0 (0.0)	177 (25.7)	689
	36-45, n (%)	1 (0.2)	1 (0.2)	9 (1.7)	21 (3.9)	6 (1.1)	0 (0.0)	131 (24.4)	537
	46+, n (%)	3 (0.8)	5 (1.3)	10 (2.6)	9 (2.3)	5 (1.3)	0 (0.0)	87 (22.7)	384
	Not mentioned, n (%)	2 (1.0)	0 (0.0)	5 (2.4)	7 (3.4)	2 (1.0)	1 (0.5)	49 (23.5)	209
**Sexual orientation,** ***P*** **value**		.10	.46	.30	.93	<.001	.99	.56	
	Homosexual, n (%)	0 (0.0)	0 (0.0)	0 (0.0)	0 (0.0)	0 (0.0)	0 (0.0)	1 (14.3)	7
	Straight, n (%)	0 (0.0)	0 (0.0)	0 (0.0)	1 (3.1)	2 (6.3)	0 (0.0)	11 (34.4)	32
	Bisexual, n (%)	0 (0.0)	5 (3.7)	6 (4.4)	6 (4.4)	9 (6.7)	0 (0.0)	36 (26.7)	135
	Not mentioned, n (%)	7 (0.4)	29 (1.4)	45 (2.2)	89 (4.4)	31 (1.5)	1 (0.1)	492 (24.3)	2026
**HIV status,** ***P*** **value**		.26	.96	.77	.47	.89	.93	<.001	
	Negative, n (%)	2 (0.7)	5 (1.7)	8 (2.8)	16 (5.6)	6 (2.1)	0 (0.0)	96 (33.3)	228
	Positive, n (%)	0 (0.0)	0 (0.0)	0 (0.0)	0 (0.0)	0 (0.0)	0 (0.0)	2 (22.2)	9
	Not mentioned, n (%)	5 (0.3)	29 (1.5)	44 (2.3)	80 (4.2)	36 (1.9)	1 (0.1)	532 (28.0)	1903
**DDF status,** ***P*** **value**		.13	.04	.04	.27	.37	.93	<.001	
	DDF, n (%)	3 (0.4)	13 (1.9)	22 (3.2)	5.6 (39)	15 (2.1)	0 (0.0)	275 (39.4)	698
	Not mentioned, n (%)	4 (0.3)	21 (1.4)	29 (1.9)	3.8 (57)	20 (1.8)	1 (0.1)	265 (17.7)	1502
**Physical appearance,** ***P*** **value**		.67	.10	.58	<.001	.21	.65	.14	
	Good looking, n (%)	1 (0.3)	10 (2.8)	10 (2.7)	44 (12.0)	10 (2.7)	0 (0.0)	91 (24.7)	368
	Not mentioned, n (%)	3 (0.2)	(24 (1.4)	41 (2.2)	52 (2.8)	32 (1.8)	1 (0.1)	449 (24.5)	1832
Total		7 (0.3)	34 (1.6)	51 (2.3)	96 (4.4)	42 (1.9)	1 (0.1)	540 (24.6)	2200

## Discussion

### Principal Findings

These findings provide insight into the representation of biases and intragroup stigma among MSM using Craigslist to seek sex with other men. Overall, very few biases were reported. This could be indicative of the unique formatting of Craigslist ads. Since there is no prescribed form for authors to complete, we found great variation in how ads were presented. In most cases, ads included very little information, resulting in limited reports of both author characteristics and biases. However, biases that were present still showed patterns and variation.

DDF bias was the most pervasive. The saliency of using the term “DDF” is consistent with previous research examining Craigslist Men Seeking Men sex ads [43]. The use of the terms “DDF,” “clean,” and “healthy” to describe people living without HIV or STIs is stigmatizing as it implies that those who are living with HIV or an STI are “diseased” and “dirty” [34,42,44]. We also found that men who described themselves as HIV negative or as “DDF” were more likely to also present a bias to be with men who are “DDF.” Similar patterns have been found in previous research; in a qualitative study examining men who posted sex ads on Craigslist, only men with an HIV negative serostatus used the term “DDF” [34]. When terms such as “DDF” and “clean” are used by MSM with an HIV negative serostatus to describe MSM living with HIV or an STI, it contributes to intragroup HIV-related stigma and can create further serostatus-based rifts within the community [16-18]. In addition, our findings showed more variation in stigma based on an author’s self-identified DDF status than any other characteristic, indicating that men who use this stigmatizing terminology may also be more likely to perpetuate other forms of stigma.

Another important finding from these data is that when men reported sexual orientation, most men identified as straight or bisexual; bias against feminine gender expression was only present in ads by these authors. This bias was only present in 1.9% of ads overall, but this finding provides insight into who is perpetuating bias against feminine gender expression. Previous research has identified a subgroup of non-gay-identified men on Craigslist who seek sex from other non-gay-identified men (who they may believe will present as more stereotypically masculine) [45-47]. The frequency of non-gay-identified men on Craigslist may be a result of the increased anonymity and invisibility of the online environment; Craigslist sex ads may be a more private way for non-gay-identified men to seek sex with men. The representation of bias against feminine gender expression may be reflective of the endorsement of hegemonic masculinity and stigma against men who deviate from expressing their masculinity in a way that is considered normative. The endorsement of hegemonic masculinity fails to recognize the fluid and nondichotomous nature of men’s gender expression [48], specifically defining masculinity in a way that is heterosexual [48-50]. Therefore, the endorsement of hegemonic masculinity exclusively by non-gay-identified MSM may indicate internalized stigma; this subgroup of men do not identify as gay, but their behaviors conflict with hegemonic masculinities because they are seeking sex with other men. While this representation of stigma may be an indication of the internalization of stigma, the bias against feminine gender expression may also be explained by a phenomenon where non-gay-identified men seek other men who are also non-gay-identified (who may be believed to present as more stereotypically masculine) because of a belief that there is a shared desire for privacy and nondisclosure about same-sex encounters [46]. Regardless of the reason behind why this subgroup presents it, this stated bias endorses hegemonic masculinity and stigmatizes those ad readers whose gender expression does not fit the stereotypical ideals of masculinity.

The presence of stigma in online sex ads may contribute to poor mental health and increased sexual risk for those who are seeking sex online. Men who have characteristics that are described in ads as undesirable may experience a fear of rejection, loneliness, and reduced self-esteem. These men may also perceive themselves as having less bargaining power when negotiating sex, possibly increasing sexual risk; previous research has examined how homophobic discrimination may influence behaviors associated with higher risk for HIV, including nondisclosure of HIV status [5,51,52] and condomless anal intercourse [11-14,53-55]. These biases may also contribute to internalized stigma among readers of the sex ads [5]. Previous research has found that internalized HIV stigma (the most prevalent form of stigma in this study) may lead to poor mental health outcomes, including depression and reduced self-esteem [3-5,56-58]. Internalized HIV stigma can also increase sexual risk-taking behaviors, including nondisclosure of HIV status to a sex partner [56,59,60] and increased drug use [60].

### Limitations

The ad authors represented in this study are limited to men who are actively seeking sex partners online. Men who seek sex partners on Craigslist differ in characteristics from men seeking partners offline [25,61,62] and may differ from men seeking partners through other online or app-based venues. Therefore, the results cannot be generalized to all MSM. Furthermore, this study analyzed Craigslist sites from 11 cities with the highest HIV prevalence in the United States; thus they may not be generalizable to cities with low HIV prevalence or to nonurban areas. We are also unable to verify the authenticity of the information posted on the ads, including the identities of the ad authors. Research indicates that online dating profiles and sex ads may misrepresent MSM demographics [63] resulting in possible misreporting of data for this study. However, despite any possible misrepresentations, we were still able to examine the representation of stigma within ads that are on Craigslist.

### Conclusions

Despite an overall low reporting of biases in ads, these findings provide insight into patterns of stigma represented on Craigslist Men Seeking Men sex ads. These findings suggest that there is a need to address intragroup stigma within MSM communities; it is important to focus on HIV-related stigma among MSM, but it is also useful to understand other forms of intragroup stigma and how they may influence mental health outcomes and sexual risk-taking behaviors, especially for MSM who are seeking sex online. Understanding patterns in the perpetuation of intragroup stigma can help to better target messages aimed at making cultural and behavioral shifts in the perpetration of intragroup stigma within MSM communities.

## Appendix

Multimedia Appendix 1 Descriptive statistics of sample characteristics.

## Abbreviations

DDF: disease and drug free
ECHPP: Enhanced Comprehensive HIV Prevention Planning
MSA: metropolitan statistical area
MSM: men who have sex with men
STI: sexually transmitted infection
