# Supplementary material for: Intragroup Stigma Among Men Who Have Sex with Men: Data Extraction from Craigslist Ads in 11 Cities in the United States
Source: JMIR Public Health Surveill. 2016 Feb 5;2(1):e4. doi: 10.2196/publichealth.4742 (PMC4869222; doi:10.2196/publichealth.4742)
Supplement: Multimedia Appendix 1 [file publichealth_v2i1e4_app1.pdf]

Appendix 1: Descriptive Statistics of Sample Characteristics by City (N=2,200)

**Characteristics (p-value)**	**MSAs with the highest HIV prevalence in the United States**					
	**New York % (n)**	**Los Angeles % (n)**	**Washington % (n)**	**Chicago % (n)**	**Atlanta % (n)**	**Miami %(n)**
**Race (P<0.001)**						
White	23.0% (46)	24.5% (49)	29.5% (59)	21.5% (43)	34.0% (68)	11.5% (23)
Black	6.5% (13)	3.5% (7)	10.5% (21)	1.5% (3)	12.0% (24)	4.5% (9)
Other	5.5% (11)	21.0% (42)	6.5% (13)	7.0% (14)	2.5% (5)	8.0% (16)
Not mentioned	65.0% (130)	51.0% (102)	53.5% (107)	70.0% (140)	51.5% (103)	76.0% (152)
**Age (P=0.003)**						
18-25	14.0% (28)	20.5% (41)	11.5% (23)	15.5% (31)	14.5% (29)	16.5% (33)
26-35	36.0% (72)	36.0% (72)	32.5% (65)	40.0% (80)	22.0% (44)	28.5% (57)
36-45	24.5% (49)	26.0% (52)	27.5% (55)	21.5% (43)	30.5% (61)	22.0% (44)
46+	17.0% (34)	8.5% (17)	17.5% (35)	16.5% (33)	21.0% (42)	23.0% (46)
Not mentioned	8.5% (17)	9.0% (18)	11.0% (22)	6.5% (13)	12.0% (24)	10.0% (20)
**Sexual Orientation (P<0.001)**						
Homosexual	0.0% (0)	0.0% (0)	0.0% (0)	0.5% (1)	0.5% (1)	1.0% (2)
Straight	4.5% (9)	2.0% (4)	0.0% (0)	2.0% (4)	1.5% (3)	0.5% (1)
Bisexual	11.5% (23)	8.0% (16)	1.5% (3)	7.5% (15)	6.0% (12)	5.5% (11)
Not mentioned	84.0% (168)	90.0% (180)	98.5% (197)	90.0% (180)	92.0% (184)	93.0% (186)
**HIV status (P<0.001)**						
Negative	9.0% (18)	25.5% (51)	15.5% (31)	12.0% (24)	12.0% (24)	10.5% (21)
Positive	0.0% (0)	0.5% (1)	0.0% (0)	0.0% (0)	0.5% (1)	0.5% (1)
Not mentioned	91.0% (182)	74.0% (148)	84.5% (169)	88.0% (176)	87.5% (175)	89.0% (178)
**DDF Status (P<0.001)**	29.0% (58)	37.5% (75)	31.0% (62)	31.5% (63)	32.5% (65)	22.5% (45)
Not mentioned	71.0% (142)	62.5% (125)	69.0% (138)	68.5% (137)	67.5% (135)	77.5% (155)
**Physical Appearance (P<0.001)**	21.0% (42)	23.5% (47)	11.5% (23)	15.0% (30)	19.0% (38)	15.5% (31)
Not mentioned	79.0% (158)	76.5% (153)	88.5% (177)	85.0% (170)	81.0% (162)	84.5% (169)

Appendix 1 Continued: Descriptive Statistics of Sample Characteristics by City (N=2,200)

**Characteristics (p-value)**	**MSAs with the highest HIV prevalence in the United States**					
	**Philadelphia % (n)**	**Houston % (n)**	**San Francisco % (n)**	**Baltimore % (n)**	**Dallas % (n)**	**Total %(n)**
**Race (P<0.001)**						
White	23.0% (46)	18.0% (36)	26.5% (53)	36.0% (72)	23.0% (46)	24.6% (541)
Black	4.0% (8)	5.0% (10)	4.5% (9)	14.5% (29)	6.5% (13)	6.6% (146)
Other	1.5% (3)	10.0% (20)	14.0% (28)	1.5% (3)	5.5% (11)	7.5% (166)
Not mentioned	71.5% (143)	67.0% (134)	55.0% (110)	48.0% (96)	65.0% (130)	61.2% (1,347)
**Age (P=0.003)**						
18-25	19.5% (39)	28.5% (57)	18.5% (37)	17.5% (35)	14.0% (28)	17.3% (381)
26-35	32.0% (64)	27.0% (54)	27.5% (55)	28.5% (57)	34.5% (69)	31.3% (689)
36-45	24.5% (49)	21.0% (42)	26.0% (52)	18.5% (37)	26.5% (53)	24.4% (537)
46+	16.0% (32)	13.0% (26)	19.0% (38)	26.0% (52)	14.5% (29)	17.5% (384)
Not mentioned	8.0% (16)	10.5% (21)	9.0% (18)	9.5% (19)	10.5% (21)	9.5% (209)
**Sexual Orientation (P<0.001)**						
Homosexual	0.5% (1)	0.0% (0)	1.0% (2)	0.0% (0)	0.0% (0)	0.3% (7)
Straight	1.5% (3)	1.5% (3)	0.0% (0)	1.5% (3)	1.0% (2)	1.5% (32)
Bisexual	5.0% (10)	1.0% (2)	6.5% (13)	6.0% (12)	9.0% (18)	6.1% (135)
Not mentioned	93.0% (186)	97.5% (195)	92.5% (185)	92.5% (185)	90.0% (180)	92.1% (2,026)
**HIV status (P<0.001)**						
Negative	8.0% (16)	13.5% (27)	18.5% (37)	8.5% (17)	11.0% (22)	13.1% (288)
Positive	0.5% (1)	0.0% (0)	0.0% (0)	1.5% (3)	1.0% (2)	0.4% (9)
Not mentioned	91.5% (183)	86.5% (173)	81.5% (163)	90.0% (180)	88.0% (176)	86.5% (1,903)
**DDF Status (P<0.001)**	25.5% (51)	36.5% (73)	38.5% (77)	30.5% (61)	34.0% (68)	31.7% (698)
Not mentioned	74.5% (149)	63.5% (127)	61.5% (123)	69.5% (139)	66.0% (132)	68.3% (1,502)
**Physical Appearance (P<0.001)**	19.0% (38)	12.5% (25)	0.00%	12.5% (25)	11.0% (22)	16.7% (368)
Not mentioned	81.0% (162)	87.5% (175)	76.5% (153)	87.5% (175)	89.0% (178)	83.3% (1,832)
